# Food Service Professionals' Perspectives on the Barriers and Facilitators to the Implementation of Nutrition Standards for School Food: A Mixed‐Methods Systematic Review

**DOI:** 10.1111/josh.70078

**Published:** 2025-09-21

**Authors:** Breda O'Mahony, Claire Kerins, Claire Barrett, Celine Murrin, Colette Kelly

**Affiliations:** ^1^ Discipline of Health Promotion, School of Health Sciences University of Galway Galway Ireland; ^2^ Tourism and Hospitality Department Munster Technological University Cork Ireland; ^3^ Health Promotion Research Centre, School of Health Sciences University of Galway Galway Ireland; ^4^ Centre for Health Research Methodology, School of Nursing and Midwifery University of Galway Galway Ireland; ^5^ Human Development and Family Studies, School of Human Ecology University of Wisconsin‐Madison Madison Wisconsin USA; ^6^ School of Public Health, Physiotherapy and Sports Science University College Dublin Dublin Ireland

**Keywords:** barriers, consolidated framework for implementation, facilitators, mixed‐methods systematic review, nutrition standards, school food

## Abstract

**Background:**

Internationally, nutrition standards for school food have been implemented. Food service professionals are key to their implementation. This mixed‐methods systematic review provides an overview of the barriers and facilitators faced by food service professionals when implementing food/nutrition standards and provides an important link between policy and practice.

**Methods:**

Peer‐reviewed and grey literature were searched across electronic databases and public health organization websites. The Consolidated Framework for Implementation Research (CFIR) was used as a framework in the analysis.

**Findings:**

Twenty‐nine studies met the eligibility criteria. The most frequently cited barriers to the CFIR constructs/subconstructs were linked to the internal school setting. This included staffing, materials and equipment, and funding. Frequently cited facilitators coded to CFIR included external partnerships/connections and staff motivation to implement the standards.

**Implications for School Health Policy, Practice, and Equity:**

Our findings highlight not only well‐documented challenges such as funding and staffing but also the novel and pivotal insights from food service professionals that point to practical, systems‐level solutions.

**Conclusion:**

Researchers and practitioners can utilize the results to devise strategies to heighten implementation, as well as capitalize on factors that aid in the implementation process.

## 1 Background

Schools play a fundamental role in promoting health and well‐being, extending their scope beyond education to create healthy food environments and provide nourishing meals for children and adolescents [1, 2, 3, 4]. Schools are uniquely positioned to promote healthy eating habits, as they can provide both nutritious meals and supportive food environments [5, 6, 7, 8, 9]. With children consuming approximately 25%–33% of their daily energy and nutrient intake through school lunches, the school food environment, and school meal provision in particular, has become increasingly important for public health intervention [10]. While some school meal programs are means‐tested, providing subsidized meals to lower‐income students, universal school meals are gaining traction as a strategy to reduce stigma and diet‐related inequalities [11].

The evidence supporting the value of structured school meal provision is robust and multifaceted. School meals are associated with improved educational attainment, attendance [12, 13], enhanced well‐being, improved health outcomes, cognitive development, and reduced obesity risk [14, 15, 16]. Additionally, school meals offer opportunities to instill healthy dietary habits and influence food preferences through the school social environment [17, 18]. Beyond health and education benefits, publicly funded school meal initiatives play a crucial role in protecting children from the immediate impacts of poverty and food insecurity [19, 20].

Numerous countries have implemented government‐sponsored or partially sponsored school meal initiatives [21, 22]. These programs typically adhere to national policies, with many countries adopting either voluntary or mandatory nutrition standards [23, 24, 25]. Such standards generally align with national and regional dietary guidelines, such as MyPlate in the United States [26], the Eatwell Guide in the United Kingdom [27], and the European Food‐Based Dietary Guidelines [28], aiming to promote healthier school food environments.

Despite widespread recognition of the advantages of nutrition standards for schools, implementation faces numerous challenges that require attention [29, 30]. An important yet frequently overlooked group in the formation and execution of school nutrient standards is food service professionals, which include food service directors, canteen staff, kitchen assistants, school nutrition coordinators, and school chefs. These stakeholders play pivotal roles in decision‐making, such as determining food selection, preparation, and production methods. Food service professionals possess unique insights into implementation barriers and facilitators [8, 31]. There has been no previous synthesis of research examining the experiences of such stakeholders with the implementation of school food nutrition standards. Understanding the policy‐to‐practice link is crucial for successful implementation. For nutrition standards to be effectively implemented, it is essential to understand how the process can be integrated into the school context [32]. This mixed‐methods systematic review explicitly explores barriers and facilitators to nutrition standards implementation as perceived by food service professionals, addressing a critical gap in the literature. While other reviews have either focused on implementation of healthy eating policies or practices more broadly [32, 33] or examined adherence to school nutrition standards [34, 35], they have not specifically examined the perspectives of those directly responsible for delivering school meal programs. School service professionals are often viewed as operational rather than strategic implementers of school food [36].

The Consolidated Framework for Implementation Research (CFIR) [37] provides a comprehensive framework for analyzing factors that influence the translation of nutrition standards into practice. By synthesizing evidence from food service professionals' experiences, this review will generate insights to help policymakers, advocates, and school administrators develop more effective strategies for implementing and sustaining school meal nutrition standards.

### 1.1 Objectives

The review objectives were to synthesize existing research regarding the perceived barriers and facilitators to the implementation of nutrition standards for school food, as experienced by food service professionals.

## 2 Methods

**FIGURE 1:**
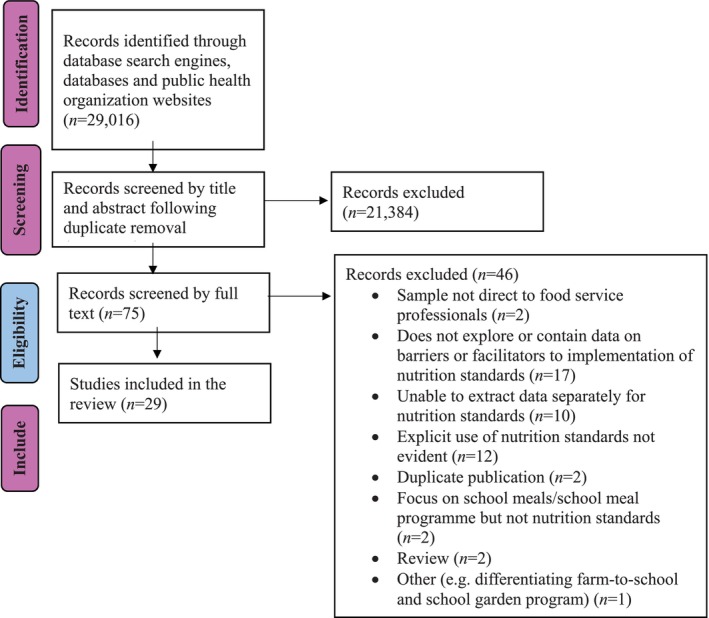
Flowchart of the literature search and review process.

The protocol, published elsewhere [38], is summarized briefly here. The review was conducted according to the Preferred Reporting Items for Systematic Reviews and Meta‐Analyses (PRISMA) guidelines [39] (Figure 1) and registered with PROSPERO, the International Prospective Register of Systematic Reviews (CRD42019117904).

### 2.1 Conceptual Framework

The CFIR was used to guide data extraction and analysis. The CFIR consists of five domains: innovation domain, outer setting, inner setting, individual domain, and implementation process domain. Due to its focus on individual‐level behavioral determinants, the protocol outlines a plan to use the theoretical domains framework (TDF); however, the pilot application of the TDF codebook on a subset of papers revealed that CFIR's broader organizational scope would better capture the multilevel factors affecting the implementation of school nutrition standards [40, 41]. CFIR is the most highly cited framework in implementation science [40, 42] and offers a pragmatic approach that allows for systematic assessment of potential barriers and facilitators across diverse contexts [43].

### 2.2 Search Criteria

The PICOS framework guided the development of the search strategy using medical subject headings (MeSH) and keyword search terms. The search strategy was customized for specific databases: CINAHL, Scopus, EMBASE, Medline, PsycINFO, and Web of Science. To identify published government reports and other grey literature, searches were conducted through Google Scholar, Open Access Theses and Dissertations, OpenGrey, RIAN, EThOS, ProQuest, WorldCat, Networked Digital Library of Theses and Dissertations, and public health organization websites. This was conducted in November 2023. Reference lists of included studies were screened, and citation chaining was undertaken (Table 1).

**TABLE 1:** Sample CINAHL title and abstract search strategy.

Search number	Search string Mh = MeSh heading
#1	recommend* OR guideline adherence [mh] OR guidance* OR protocol* OR nutrition policy [mh] OR strateg* OR standard* OR nutri* OR health promotion [mh]
#2	school lunch* OR school meal* OR canteen* OR food services [mh] OR school food OR menu planning [mh] OR food program* OR school* meal* program* OR school dinner*
#3	school* OR education*
#4	#1 AND #2 AND #3

### 2.3 Study Eligibility

Primary research studies using qualitative, quantitative, or mixed methods approaches were eligible for inclusion. Studies were included if they examined barriers and facilitators to the implementation of nutrition standards for school food as experienced by food service professionals. Table 2 presents the detailed eligibility criteria according to population, intervention, types of food, study setting, focus, and study type. No restrictions were imposed on publication year or language.

**TABLE 2:** Study eligibility criteria.

	Inclusion	Exclusion
Population	Food service professionals, that is, catering management and staff, school principals/managers, contracted catering suppliers, food service directors and managers, program coordinators/administrators, and procurement staff.	Demand side stakeholders, that is, children/students' and parents'/guardians' experiences and perceptions.
	Studies from officials and or government organizations that may influence food provision in schools (e.g., policymakers).	Teachers, school administrators, school management/leadership, and school staff not directly involved with implementing the school meals standards.
Intervention	School meal standards	Health‐promoting schools
	Nutrition standards for school meals	School nutrition policies
	School meals program	Healthy eating initiatives/interventions
	Food and nutrient standards	Unless such policies and interventions are based on school meal nutritional/food standards.
	*These can be voluntary or mandatory	
Types of food	Any/all meals, beverages, and snacks provided in schools, including breakfast clubs and vending machines.	Food consumed/purchased outside the school environment.
Study setting	Educational establishments where, typically speaking, students aged 5–18 years attend (i.e., primary/elementary/junior high/middle/post primary/secondary/senior and high school).	Preschool, postsecondary school, and any third‐level settings.
		Implementation of school meals during a pandemic (e.g., COVID‐19) and outside of the traditional school calendar (e.g., summer holidays).
Focus	Stakeholders' perceptions/experiences/feedback on school food standards.	Student experiences of implementation of standards
Study type	Primary research studies (from gray or primary‐reviewed literature), including: Qualitative studies: case studies, grounded theory, ethnography, action research studies, and case studies Quantitative studies: case control studies, quasi‐experimental studies, randomized controlled trials, and cross‐sectional studies Mixed methods (combining qualitative and quantitative methods of data collection and analysis); focus groups, interviews, surveys, questionnaires, and observation.	Editorials, commentary, reviews, and opinion pieces.

### 2.4 Study Selection and Appraisal

Two independent reviewers (B.O.M. and C.B.) conducted the study selection process through title/abstract screening and full‐text review. To ensure rigor, both screening processes were piloted before formal screening began. Discrepancies between reviewers were resolved through discussion and consensus with the research team. The methodological quality of included studies was assessed using the Mixed Methods Appraisal Tool (MMAT). No studies were excluded based on quality scores.

### 2.5 Data Extraction and Synthesis

Data extraction was conducted independently by two reviewers (B.O.M. and C.Kelly) using a standardized form. Discrepancies in extraction were resolved through discussion until consensus was reached. One reviewer (B.O.M.) led the synthesis process using deductive analysis, with regular consultation with review team members (C.K. and C.Kelly). Following data familiarization, one reviewer (B.O.M.) conducted deductive coding using the CFIR codebook, with coding decisions regularly reviewed by the team. Coded barriers and facilitators were then rank‐ordered based on frequency of mentions across studies to identify prevalent factors influencing the implementation of nutrition standards for school food.

## 3 Findings

### 3.1 Study Selection

Post removal of duplicates, the search yielded a total of 21,459 records. Following title and abstract review, 75 records were included in the full text review. Following full text review, 29 studies were included (Figure 1).

### 3.2 Study Characteristics and Quality Appraisal

Study characteristics are provided in Table 3. Included studies largely used qualitative data collection methods (*n* = 17), followed by mixed methods (*n* = 8) and quantitative methods (*n* = 4). The majority of studies were located in the United States (US) (*n* = 17), followed by Australia (*n* = 5), Canada (*n* = 3), Norway (*n* = 2), Uruguay (*n* = 1), and England (*n* = 1). In terms of study quality, using the MMAT, the majority of studies were rated as average to high quality.

**TABLE 3:** Characteristics of included studies (*n* = 29) that explored barriers and facilitators to the implementation of nutrition standards for school food.

First author, year, country of origin	Publication type	Design	Methods, data source	Study participants (*n*)	Setting	Intervention type	Types of food	Voluntary or mandatory	MMAT score (%)
Asada, 2016 [44], USA	Article	Qualitative	Multiple case study approach interviews	School professional (*n* = 37); 11 districts' food service directors (FSD), 8 athletic directors/PE teachers/boosters, 7 principals/vice principals, 5 cafeteria managers, and remaining 8 respondents were a nurse, consumer science/health teacher, technical assistance provider, and finance administrator	High schools (*n* = 9) across 8 states	USDA (United States Department of Agriculture)	School snacks	Mandatory	72
						Smart Snacks Nutritional Standards			
Asada, 2017 [45], USA	Article	Qualitative	Semi‐structured telephone interviews	FSDs (*n* = 9) from high schools that had achieved Healthier US Schools Challenge	High schools (*n* = 9)	Nutrition Standards in the National School Lunch and Breakfast Programs	School meals	Mandatory	72
Vine, 2013 [46], Canada	Article	Qualitative	Semi‐structured interviews (*n* = 22)	Local public health unit and community agencies with mandates supporting school nutrition (*n* = 8) and school‐level participants (secondary school principals, vice principals, teachers, and admin) (*n* = 14)	Secondary schools (*n* = 9)	School Food and Beverage Policy/Program Memorandum No. 150 (PPM 150)	School food and beverages	Mandatory	72
Pettigrew, 2012 [47], Australia	Article	Mixed methods	Semi‐structured survey	School principal online survey (*n* = 310), school principal interview (*n* = 10)	Primary schools (*n* = 6), secondary school (*n* = 3), combined (*n* = 1)	Healthy Food & Drink Policy	School food and beverages	Mandatory	29
Girona, 2018 [48], Uruguay	Article	Mixed methods	Semi‐structured interviews and surveys	Principals (*n* = 59)	Primary schools (*n* = 38), secondary schools (*n* = 21)	Healthy Snacking Initiative	School snacks	Voluntary	72
Ardzejewska, 2012 [49], Australia.	Article	Mixed methods	Quantitative audit approach, follow‐up semi‐structured interviews	Principal or deputy principal and canteen manager	Primary school (*n* = 2), secondary school (*n* = 2)	Healthy School Canteen Strategy using The Canteen Menu Planning Guide	School food and beverages	Mandatory	42
Farris, 2021 [50], USA	Article	Qualitative	Semi‐structured interviews	School nutrition directors (SNDs) (*n* = 10)	No school was mentioned—all SNDs in one southeastern state were invited to participate interview	National School Lunch Program by the USDA	School meals	Mandatory	100
Holthe, 2011 [51], Norway	Article	Qualitative	Multiple case design involving questionnaire, interviews, observation survey, and focus group interviews	Participants included school principals, project leaders, teachers, and students.	Three secondary schools in Norway selected as case studies	National Guidelines for Healthy School meals	School meals	Voluntary	85
				*Student data not used					
Cornish, 2016 [52], USA	Article	Mixed methods	Qualitative semi‐structured telephone interviews and quantitative online questionnaire	FSD (*n* = 67) participated in the telephone survey and an online survey completed by *n* = 57 respondents; food service director (*n* = 43), head cook (*n* = 16), kitchen manager (*n* = 6), dietary manager (*n* = 5), secretary (*n* = 2), administrator (*n* = 1) and bookkeeper (*n* = 1)	Rural schools	National School Lunch Program	School meals	Mandatory	29
Tabak, 2015 [53], USA	Article	Qualitative	Semi‐structured interviews	FSDs (*n* = 8) from school districts in a single region in Missouri	Primary school (*n* = 8)	National School Lunch Program	School meals	Mandatory	72
McPherson, 2021 [54], Australia	Article	Qualitative	Semi‐structured interviews (*n* = 12); 10 individual and 2 focus groups	Principals (*n* = 4), assistant principal (*n* = 1), canteen managers (*n* = 5), food services manager (*n* = 1) and canteen staff members (*n* = 3)	Primary (*n* = 5), primary and secondary (to year 12) (*n* = 1)	Healthy school canteen policy based on the Australian Dietary Guidelines	School canteen food	Voluntary	72
Potter, 2011 [55], USA	Article	Mixed methods	Qualitative and quantitative methods; interviews, focus groups, questionnaires, and logs	Five out of 25 schools participating in the pilot program were selected for the evaluation	Schools (*n* = 5); 1 urban elementary, 1 rural elementary, 1 suburban middle, 1 rural high, and 1 rural junior/senior high school	Mississippi Fruit and Vegetable Pilot Program to schools participating in the National School Lunch Program	Fruit and vegetables in school meals	Pilot Program	14
		Pross evaluation		Participants; program staff (*n* = 11) and administrators (*n* = 6) via interviews and logs; student (*n* = 42) and parent (*n* = 19) focus groups; student questionnaires (*n* = 660); and school staff questionnaires (*n* = 207)					
				*Student and parent data not used					
McIsaac, 2019 [56], Canada	Article	Quantitative	Online survey	School principals or appropriate designated individual with experience in school food service	Schools (*n* = 237); elementary grades (*n* = 170), junior high grades (*n* = 85), high school grades (*n* = 56)	School food and beverage	School food	Mandatory	72
Day, 2015 [57], England	Article	Qualitative	Interviews	Catering managers (*n* = 6) and head teachers (*n* = 5) preparing for implementation of the Universal Infant Free School Meal	Primary Schools (*n* = 8)	Universal infant free school meal	School meals	Mandatory	100
Holthe, 2011 [58], Norway	Article	Longitudinal	Multiple case design using an explorative approach	School principals, project leaders, students, and teachers	Secondary Schools (*n* = 3)	The Norwegian national guidelines for health school meals	School meals	Voluntary	85
			Interviews with school principals and project leaders	*Student data not used					
			Focus group interviews with students and teachers						
Stang, 1997 [59], USA	Report	Quantitative	Survey	Food service personal (*n* = 628) comprised of district food service director (*n* = 113), school food service manager (*n* = 135), cook/manager (*n* = 339), food service assistant (*n* = 32)	Elementary school (*n* = 339), junior high/middle School (*n* = 59), high school (*n* = 63), K‐12 school (*n* = 155), other school (*n* = 12)	School meals initiative for healthy children following the US Dietary Guidelines	School meals	Voluntary	85
Asada, 2020 [9], USA	Research article	Qualitative	Semi‐structured interviews	Food service directors (*n* = 20), principals (*n* = 13), school nurses (*n* = 2), health education teachers (*n* = 2), and wellness department coordinator (*n* = 1)	High schools (*n* = 22)	Nutrition Standards in the National School Lunch and Breakfast Program	School lunch and breakfast programs	Mandatory	100
Roberts, 2009 [60], USA	Journal article	Qualitative	Semi‐structured interviews	Principals (*n* = 24) and FSDs (*n* = 10)	High school (*n* = 24)	Texas Public School Nutrition Policy	School meals and beverages	Mandatory	57
Nollen, 2007 [61], USA	Journal article	Qualitative	Semi‐structured interviews	Principal (*n* = 8), dietician/food service manager (*n* = 7)	High schools (*n* = 8)	National School Lunch Program and School Breakfast Program meet USDA nutrition guidelines	School food and beverage	Mandatory	85
Eddie, 2020 [62], USA	Article	Mixed methods	Quantitative and qualitative methods; surveys and on‐site observations	Two‐part survey: principals (*n* = 6) and food service workers (*n* = 14) completed part 1	Elementary and middle schools (*n* = 6)	Nutrition standards for school meals and all other foods and beverages sold in the school mandated by the Healthy Hunger‐Free Kids Act of 2010	School meals and all other foods and beverages sold in the school	Mandatory	29
				Food service workers (*n* = 14) completed part 2					
				Observational study: record availability of vending machines, types of snacks or beverages sold, location of vending machines, and all other alternative food & beverage sources. File notes were also taken to gain insight into the school environment					
				*Observational data not used					
Brouse, 2009 [31], USA	Article	Quantitative	Self‐report questionnaire	Food service directors (*n* = 297)	Elementary, junior high, and high schools	National School Lunch Program—following USDA guidelines	School meals	Voluntary	72
United States Government Accountability Office [63]	Government Report	Mixed Methods	Qualitative and quantitative; site visit, interviews, survey, document review, and financial analysis	The relevant methodology for this is: national survey with nutrition directors in 50 states	Elementary, middle, and high schools	National School Lunch Program	School meals	Mandatory	72
				Site visits (*n* = 8) and interviews with the nutrition directors who oversee these districts					
				To gather further information, interviews with representatives from several stakeholder groups, including a group of eight SFA directors representing both their own districts and their regions of the county, and a group of 11 relevant industry representatives					
Reilly, 2017 [64], Australia	Brief Report	Quantitative	Survey	Canteen managers (*n* = 184)	All primary schools (enrolling children aged 5–12 years) having an operational canteen (*n* = 265)	Healthy Canteen Policy based on traffic light system	Canteen	Mandatory	72
Mâsse, 2013 [65], Canada	Article	Qualitative	Semi‐structured interviews	50 school informants (principals *n* = 11, teacher/school informants *n* = 33; classroom teachers *n* = 21, PE specialist *n* = 9, cafeteria staff *n* = 3 and home economics teacher *n* = 3)	School (*n* = 17): elementary (*n* = 10), junior high school (*n* = 1), senior high school (*n* = 1), high schools (*n* = 5)	Food and Beverage Sales in Schools guidelines based on Canada's 2007 Food Guide	Food and beverages	Mandatory	85
Abery, 2014 [66], Australia	Journal article	Qualitative	Case study	School A: Principal (*n* = 1), canteen manager (*n* = 1), parents (*n* = 6), students (*n* = 8)	Primary schools (*n* = 2)	Rite Bite Healthy Food and Drinks strategy for South Australian Schools and Preschools	Food and drink	Mandatory in all South Australian schools	85
			Focus groups, semi‐structured interviews, and observation	School B: Principal (*n* = 1), canteen manager (*n* = 1), parents (*n* = 6), students (*n* = 28)					
				*Only data from principal and canteen managers used					
Rida, 2019 [4], USA	Journal article	Mixed Methods	Convergent parallel mixed methods study; qualitative and quantitative data were collected in parallel, analyzed separately, and then merged	Surveys with school food‐service directors/managers (*n* = 260) (cashiers *n* = 4, cooks *n* = 34, cafeteria staff *n* = 7, food service directors *n* = 60, kitchen staff *n* = 27, managers *n* = 97, others *n* = 26)	School food service personnel across Nebraska	USDA nutrition standards	School meals	Mandatory	85
				Focus groups (*n* = 15) (school food‐service managers *n* = 10, head cooks *n* = 3 and directors *n* = 2)					
Chu, 2012 [67], USA	Research Brief	Qualitative	Focus group	Eight focus groups with school food service personnel (*n* = 67)	School food service personnel from five states, including rural, suburban, and urban schools	Dietary Guidelines for Americans	Whole‐grain foods in school meals	Unknown	72
Habayeb, 2013 [68], Waterloo Region	Master's thesis	Qualitative	Semi‐structured interviews	Food service participants consisted of managers (marketing, sales, business development, general), owners, employees, a president, and a director of nutrition.	Food service organizations selling food to the Waterloo Region schools (*n* = 18)	Ontario School Food and Beverage Policy P/PM 150	School Food and Beverage	Mandatory	100
Summers, 2013 [69], Baltimore	Doctorate dissertation	Qualitative	Semi‐structured interviews (*n* = 19)	Food service personnel included food and nutrition service directors (*n* = 8), district dietitians (*n* = 3), nutrition or program coordinators (*n* = 4), quality control specialists (*n* = 2), and other district‐level personnel (*n* = 2)	Food service personnel from districts and those implementing Meatless Monday	National School Lunch Program	Vegetarian School Meals	Mandatory	100

*Note:* Setting refers to the physical school type in which the nutrition standards for school food are operating. Intervention type refers to the specific type of program, strategy, intervention, or initiative implemented to improve or change some aspect of the school food provision. Voluntary or mandatory refers to how the implementation of nutrition standards for school food is structured. Mandatory standards are required by law, regulation, or policy, often at the national or regional level. Voluntary standards are generally adopted at the discretion of the school or region.

### 3.3 Barriers and Facilitators

A summary of perceived barriers and facilitators coded to the CFIR constructs is presented in Table 4 with supporting data (quotes/narrative summary) included. A narrative summary of the most frequently cited barriers (those discussed in seven or more included studies) and facilitators (those appearing in five or more studies) is presented below.

**TABLE 4:** Summary of perceived barriers and facilitators to the implementation of school meal standards coded to the CFIR.

CFIR domains	CFIR construct	CFIR sub‐construct	Papers identified barriers	Papers identified facilitators	Supporting data (quotes/narrative summary)
I. Innovation Domain	A. Innovation Source		2	0	Barrier: Several respondents felt that more people should have been involved in policy development. The FSDs especially felt that their expertise was ignored [60].
	B. Innovation Evidence Base		1	0	Barrier: It would be nice to see some research, or if someone could present empirical results… Teachers are quite skeptical, so whenever something new comes along, it's difficult and should preferably have been thoroughly tested before we throw ourselves into it [58].
	C. Innovation Relative Advantage/Disadvantage		1	1	Barrier: High school principal “it's great to be promoting healthy food, on the one hand it's, you know, our athletic program half the funding we used to have for running our athletic program was coming from the profits.” [65] Facilitator: One principal stated, “I think any standard that people feel would be in the best interest of teenagers…I would be for it. If they feel there is something that would help students with their health and fitness and be able to control obesity, I would certainly be in favor of it.” [61]
	D. Innovation Adaptability		2	2	Barrier: We can't follow it all the time; we need “one time exceptions” “So what was at the grad barbecue? Well, there was regular coke and stuff like that. They wouldn't fall under the guidelines, so. We just kind of ignore the guidelines and because, if we brought in juices and stuff like that, probably wouldn't be drunk at an event like that.” (Middle/high school principal) [65] Facilitator: “I'm hopeful that these flexibilities will become basically a permanent part of the program.” [50]
	E. Innovation Trialability		0	0	
	F. Innovation Complexity		2	0	Barrier: Principals and canteen managers claimed that many of the terms used within Right Bite were “confusing,” “fuzzy,” and “difficult to interpret.” [66]
	G. Innovation Design		7	4	Barrier: We found it difficult to understand the scope of the guidelines (just vending or all food served) and where they apply (fundraising activities, activities organized by Parent Advisory Council, bake sale items, classroom treats, outside school hours activities, advertising in school, accepting sponsorship from companies, and accepting incentives from vending machines company) [65] Facilitator: Having provincial resources helped us with implementation (e.g., booklet provided by the Ministry of Education, website, and the Fruit and Vegetable program) [65].
	H. Innovation Cost		2	0	Barrier: Principal stated “I'm torn…I'm mandated to do more instruction, I'm mandated to move all of these kids…and I'm just getting another mandate that I can't foot the bill on. To do it right, I need more resources…You can't throw me a wellness program and say, but there's no money, and that's what we continually see from the government.” [61]
II. Outer Setting	A. Critical Incidents		0	0	
	B. Local Attitudes		0	0	
	C. Local Conditions		3	1	Barrier: There was a perceived lack of support for other schools in the region that had not previously had a kitchen, and it was foreseen that they would struggle because of limited capacity without much support [57]. Facilitator: Having local suppliers that comply with the guidelines is necessary [65].
	D. Partnerships and Connections		0	11	Facilitator: Information materials provided by the DET were reported to be helpful in explaining the Policy to school staff, canteen managers and parents [47].
	E. Policies and Laws		5	5	Barrier: School principals said that they found it difficult to enforce canteen managers to comply with the requirements of the law as the law does not establish any type of sanction [48]. Facilitator: As a Texas FSD said, “Schools make decisions based on policy”. So as long as there's a policy that is within hands' reach, so you can actually print it out and put it on [44].
	F. Financing		3	2	Barrier: Delays in funding receipt from the Local Authority with only 8 weeks to go, and another school perceived that some local schools would miss out on funding that they had bid for because it was being spread too thinly [57]. Facilitator: Some directors received financial support from their district; these districts were willing to invest additional resources in improvements to food service such as enhancements to the cafeteria or kitchen spaces [53].
	G. External Pressure				
		1. Societal Pressure	0	0	
		2. Market Pressure	0	0	
		3. Performance Measurement Pressure	0	1	Facilitator: The HUSSC: SL award brought positive recognition and boosted the reputation of any food service department that attained it [44].
III. Inner Setting	A. Structural Characteristics				
		1. Physical Infrastructure	3	1	Barrier: The schools lacked an area for the canteen's basic functions of production, sales, and eating areas [51]. Facilitator: School B's canteen had a separate preparation and serving area, which meant that food preparation did not need to be disrupted during recess time [66].
		2. Information Technology Infrastructure	0	1	Barrier: Training on computer diet analysis [59].
		3. Work Infrastructure	14	0	Barrier: School food service staff in all eight districts noted that workload increased primarily because of the need to prepare more fruits and vegetables each day to meet requirements [63].
	B. Relational Connections		1	2	Barrier: “Last year, we organized it in a way that each group taking home economics worked in the canteen. But it wasn't voluntary and everyone had to do it. So the effort level was only so.” [51] Facilitator: Directors also recognized the benefits of their relationships with and support from staff [53].
	C. Communications		1	1	Barrier: Feelings of isolation were reiterated by the canteen manager in school B who felt ‘separate from the school’ and not in control. All of the major decision‐making was taken away from her and she claimed: They don't really keep me informed… you know they keep telling me it's running at a loss, that's all they keep telling me [66]. Facilitator: The most salient internal partnership cited by all respondents was with school leadership; as the “go‐to person” and “hub of communications,” the principal was a critical stakeholder [44].
	D. Culture				
		1. Human Equality Centeredness	0	0	
		2. Recipient Centeredness	1	1	Barrier: All four schools had a majority of Muslim students who required Halal products. The audit revealed that each school sold the same brand of Halal pies and sausage rolls (identified as red foods). “Yes it is a red, but how do you get around not selling that” (canteen manager) [49].
		3. Deliverer‐Centeredness	1	0	Barrier: A reluctance to engage with the Policy by a small number of canteen staff [47].
		4. Learning Centeredness	0	0	
	E. Tension for Change		1	0	Barrier: Respondents revealed the tension between, on one hand, providing nutritious foods to students and, on the other, the revenues that schools gain as a result of selling unhealthful foods [46].
	F. Compatibility		9	1	Barrier: Across the cases, all teachers identified conflict between allocating time for their educational aims, on the one hand, and implementation of the guidelines, on the other [51]. Facilitator: “I think it is pretty well established and… being implemented very well in our district.” [60]
	G. Relative Priority		3	0	Barrier: Principals felt student health was important but most did not consider it the school's top priority [61].
	H. Incentive Systems		2	0	Barrier: The ability to recruit school nutrition professionals to rural communities due to unique rural lifestyles and poorer compensation compared to urban positions [9].
	I. Mission Alignment		2	2	Barrier: Although attempting to create a healthy school canteen, School A explained that profit remained a higher priority than health. School A, “I mean there's no point in selling healthy food if it's not going to make money because at the end of the day I've got to be able to pay my bills as well” (canteen manager) [54]. Facilitator: Our job is to facilitate learning, and healthy food is an important means of helping the students to learn more. And certainly, in a larger perspective, we are helping to create healthy young people [58].
	J. Available Resources				
		1. Funding	11	5	Barrier: FSDs in New York and Iowa reported challenges with finding whole grain‐rich products that were affordable: We have had great success with our vendors and purveyors giving us a wide variety of products. But the cost has been sometimes 25% higher and that has really prohibited what we can serve to the kids [45]. Facilitator: On the other hand, the FSDs in California experienced no challenges with this component, stating: I haven't seen a significant increase in cost [45].
		2. Spaces	8	0	Barrier: In secondary public schools, there was the lack of an adequate place to prepare homemade food. Many canteens had only a small place that enabled them only to sell packaged products [48].
		3. Material and Equipment	13	1	Barrier: One school had no functional kitchen for weeks and had to serve packed lunches daily for children over the summer term [57]. Facilitator: The smaller schools had only had to make few amendments such as equipment updates because maximum school meal uptake was still only perceived to be small and thus manageable [57].
	K: Access to knowledge and information		7	5	Barrier: Results also showed that there was no school‐wide training to facilitate implementation at any of the case schools [58]. Facilitator: School E's Food Services Manager demonstrated great awareness of online resources and programs enabling the correct implementation of the policy [54].
IV. Individual Domain	A. High‐level Leaders		1	3	Barrier: School principals said that eating habits were just one of the many problems they had to deal with and did not seem to invest additional time or effort to address them [48]. Facilitator: Directors felt that his/her leadership, positive attitude, and ability to be flexible were particularly important as s/he was seen to set the tone for the department [53].
	B. Mid‐level Leaders		0	1	Facilitator: Teacher support for SNP is a vital component of their promotion and successful implementation at the school level [46].
	C. Opinion Leaders		1	0	Barrier: Four were against the policy and thought that it should be “done away with.” [60]
	D. Implementation Facilitators		0	0	
	E. Implementation Leads		0	0	
	F. Implementation Team Members		0	0	
	G. Other Implementation Support		0	0	
	H. Innovation Delivers		0	0	
	I. Innovation Recipients		0	0	
	Characteristics sub‐domain				
		A. Need	0	1	Facilitator: We needed this “Well I think it's, it's late in coming but it's about time, yeah” (Middle/high school teacher) [65]
		B. Capability	4	1	Barrier: Some directors reported that staff tasked with food preparation and service initially expressed low self‐efficacy for the new food preparation requirements [53]. Facilitator: “You don't have to be a food consultant to know that the freshly produced food is better than the preserved packaged food” (Principal) [54]
		C. Opportunity	9	1	Barrier: “We may get in a whole grain rich product that either appearance wise or texture or taste or mouthfeel just you know isn't working really well for us and we'll have to you know try to identify another product.” [50] Facilitator: Time, in terms of the time to develop new products acceptable to students and the time needed to implement new nutrition standards, played a large role in SNDs ability to meet acceptable implementation changes to school meal programs [50].
		D. Motivation	2	7	Barrier: One dietitian said, “actually we have to break the cook's resistance most of all,” which she noted is especially true among the older staff. A few participants indicated that cafeteria staff and school administration are not always eager to eat vegetarian meals or try a new food item [69]. Facilitator: “Um for the most part I think the changes are wonderful. Um, I'm behind the uh, fruits and vegetables, I think it is been a long time coming, and uh, the whole grains are great.” [52]
V. Implementation Process Domain	A. Teaming		0	2	Facilitator: Challenges related to extra time for snack preparation were solved with assistance from additional school staff and students or by ordering prepackaged snacks [55].
	B. Assessing Needs				
		1. Innovation Delivers	0	0	
		2. Innovation Recipients	1	0	Barrier: According to the interviews, although the initiative encourages availability of healthy foods, children and adolescents still prefer unhealthy products, which makes it difficult to convince canteen managers to change their offer of foods and beverages [48].
	C. Assessing Context		0	0	
	D. Planning		0	1	Facilitator: This FSD described proactive implementation as a key factor in his success: “Doing the right thing before the right thing is necessary.” While these FSDs still faced challenges during the most recent school meal reform efforts, such earlier efforts may have led to fewer obstacles during the most recent implementation compared to schools that had not initiated any changes [45].
	E. Tailoring Strategies		0	2	Facilitator: Schools worked with produce providers to improve delivery timing, but also had backup strategies. One school occasionally served more than one fruit or vegetable snack a day if one was close to spoiling. Many schools served dried fruit on the few occasions when fresh produce was not available [55].
	F. Engaging				
		1. Innovation Delivers	5	5	Barrier: The head teacher advocated that the scheme should have looked at individual school capacity and the potential for schools to support each other in the same region [57]. Facilitator: To increase the likelihood of their external caterer continuing to be compliant, School C removed canteen rent fees [54].
		2. Innovation Recipients	1	0	Barrier: Although secondary public schools should have a canteen committee composed of teachers that controls what is sold in the canteens, they recognized difficulties to make this committee work and stated that they usually do not have much influence on the decisions of canteen managers [48].
	G. Doing		0	2	Facilitator: Having to run trials in preparation [57].
	H. Reflecting and Evaluating				
		1. Implementation	0	0	
		2. Innovation	0	0	
	I. Adapting		3	1	Barrier: While directors reported understanding the importance of presenting foods in ways that made them appealing to students, many reported significant limitations, particularly with regard to cafeteria space for display. Directors tried to address this by altering their menu planning [53]. Facilitator: Would have to rethink the structure of the school day to accommodate increased turnover [57].

*Note:* Empty cells indicate that no barriers or facilitators were identified for those constructs.

### 3.4 Barriers

Irrespective of the country/regional variations, several barriers were identified that hindered the implementation of nutrition standards for school food, with the majority originating from within the internal school environment. One of the most commonly cited barriers was staffing [9, 46, 47, 50, 51, 52, 53, 56, 57, 58, 63, 66, 69]. Due to food service professionals being unable to offer nutritious food that meets the policy's nutritional requirements at an affordable cost, schools face the risk of losing revenue. A school's canteen revenue has implications for food service professionals' jobs [46, 51, 52, 53, 56, 57, 58, 66]. Furthermore, some schools lacked sufficient staff to run the canteen and cover employee expenses, leading to reliance on unpaid student help for tasks such as food preparation, sales, and cleaning [51, 58]. The barrier also encompassed an increased workload for food service professionals in preparing food that met the school food requirements, which negatively affected implementation [8, 47, 53, 59, 63, 68]. In rural schools, limited staff capacity resulted in staff taking on multiple roles, impacting their ability to apply for grants to fund equipment or initiatives that could support school meal standards implementation [9, 57]. Another obstacle experienced by food service professionals was a lack of space for preparation, serving, and storage [31, 47, 48, 53, 55, 63, 66, 69], which limited many canteens to using only packaged products [48] and resulted in more frequent food deliveries, increasing costs from suppliers [63].

Other frequently cited barriers to implementation experienced within the internal school setting included a lack of resources in the form of funding materials [31, 45, 47, 52, 53, 59, 60, 63, 66, 67, 69] and equipment [31, 46, 50, 51, 53, 56, 57, 63, 66, 67, 68, 69]. This encompassed the inability of food service professionals to source affordable products that meet the policy nutritional requirements [31, 45, 52, 59, 63, 66, 67, 69], and manage increasing food costs [47, 53, 66]. Identifying and procuring policy‐compliant food options was also a difficulty experienced by food service professionals [46, 51, 53, 63, 67, 69]. Equipment was often inadequate, with some held together in an effort to keep them operating [50, 56].

Another impediment within the internal school setting was accessing training and guidance [31, 45, 51, 54, 59, 69]. This included a lack of school‐wide training to facilitate the implementation of standardized menus, computer diet analysis training, and menu creation [51, 58]. It was also noted that when training sessions were provided, food service managers often had a desire to attend but were unable to do so due to time constraints [53, 66]. Resources directed to schools resulted in minimal engagement due to food service professionals' lack of time [66]. The lack of time also impacted preparation time for creating high‐quality meals, which sometimes resulted in canteens utilizing time‐saving shortcuts [50] such as limiting menu choices and buying pre‐chopped salad ingredients instead of using scratch cooking techniques with fresh produce [66].

Barriers identified by individuals directly or indirectly involved in the implementation of the school food standards included a lack of district support [62] and inadequate communication regarding the policy [48]. The short timeline from passing nutrition standards to implementation [50, 52, 63], along with differences in calorie range requirements between the 6‐grade and 8‐grade groups and the 9‐grade and 12‐grade groups, created challenges in adopting menus that met both grade groups' requirements [63].

Factors relating to the external school environment included the burdensome nature of the policy that often lacked input from experts [60, 61]. Food service professionals felt that the policy was developed using a top‐down approach and should have included the stakeholders who implement school meals [60, 61]. This is directly linked to the barriers to implementation of such school nutrition standards. The standards were considered problematic [31, 48, 49, 52, 63, 65, 69], with the classification of products noted as being challenging and confusing, as products seen as acceptable were not and vice versa [49, 69], This also extended to understanding the scope and application of the nutrition standards [65], as well as the limited explanatory materials about the guidelines [48].

### 3.5 Facilitators

Across all counties/regions, partnerships and collaboration in the external school environment were one of the most frequently reported facilitators in the implementation of nutrition standards for school food [8, 9, 44, 47, 51, 53, 54, 55, 65, 67, 69]. This included having local suppliers that comply with the standards, which was seen as essential [65]. For rural food service professionals, this collaboration mitigated procurement barriers due to geographic location, as neighboring districts joined and formed purchasing co‐ops [9]. Similarly, to mitigate storage issues, schools transferred produce to other nearby schools [55]. Schools that received external support appeared more confident in their menu selection [54], and food service professionals believed it helped them to overcome impediments related to limited staff and infrastructure [47]. The external support came from community‐based organizations [44, 54] and state departments that provided hands‐on technical assistance and resources [44, 47]. Partnerships also existed in the form of peer networks with partner school districts, which facilitated the sharing of best practices among peer food service professionals' [44].

External to the school setting, policy underpinned the nutrition standards and therefore served as an important facilitator to implementation [44, 47, 48, 65, 69]. Food service professionals perceived that policy gave legitimacy and authority to make changes that may otherwise have been impossible at the school level [45, 47, 48, 49, 50, 51, 52, 53, 54, 55, 56, 57, 58, 59, 60, 61, 62, 63, 65, 66, 67, 68, 69]. Within the internal school setting, knowledge of and guidance about the standards alleviated fears regarding the implementation of the standards [53]. This access to information helped bring everyone on board with the successful implementation of standards [45, 53].

Motivation was a prominent facilitator for individuals involved in the implementation of nutrition standards [50, 52, 53, 54, 55, 61, 65]. Food service professionals prioritized health over profit; indeed, even when operating at a loss, they valued the provision of healthy meals [54]. Some noted successes, including students trying fruits and vegetables, as well as other foods that were new to them [55]. The enthusiasm of food service professionals stemmed from their strong identification with the nutrition standards for school food and viewing these standards as essential [52, 53]. A sense of pride motivated some food service professionals' who were pleased to work in school nutrition, satisfied with the guidance received at the state level, and felt a sense of accomplishment in being ahead of other U.S. states in their successful implementation [50].

## 4 Discussion

Using a conceptual implementation framework, this review identified a range of key barriers and facilitators to the implementation of school food standards, with a particular focus on the often‐overlooked, yet crucial perspectives of food service professionals. The findings also highlight the complexity of implementation, with barriers predominantly evident within both the internal school environment and the external context of schools. Some factors pertained to characteristics of individuals, features of the school meal standards, and the process of implementation. Interrelationships between factors operating across multiple levels and settings created some factors that were both barriers and facilitators to implementation and emphasized the complex and dynamic nature of the implementation of such school food standards. While the majority of included studies were based in the United States, the consistency of themes across diverse settings suggests common challenges in the implementation of nutrition standards for school food. In this review, we did not identify substantial differences in barriers or facilitators across countries or regions. Stronger institutional support for school meals was identified in Norway, which may reduce the local‐level implementation challenges reported in the US‐based studies [58]. However, the factors appeared to be broadly consistent regardless of the geographic context. Similarly, we did not identify consistent differences in the types of barriers or facilitators reported across food service professionals' roles.

One of the most frequently cited barriers identified in this review was the lack of adequate funding, which aligns with previous research on policy implementation in schools [70, 71, 72, 73, 74]. Financial investment is essential for materials, equipment, and supporting the increased cost of healthy foods. It also extends to recruitment of staff wages, and training. The removal of competitive foods that did not meet the school food standards reduced school revenue and had effects on other aspects of school operations. This revenue often releases funds for curricular and extracurricular activities [60, 65]. Governments must recognize the need for financial support when creating policies and release funds with sufficient time for effective planning. Meshkovska et al. [72] call for a whole‐of‐society approach with stronger cooperation across all governmental sectors to enable stakeholders to deal with these challenges. However, what is unique in this review is the strategic role that food service professionals play in navigating and adapting to these constraints. What this often underrepresented group of food service professionals knows is often overlooked. Their experiences through cross‐role collaboration, innovation, and flexibility not only show what hinders implementation but also that they are active problem solvers to sustain school meals. Many described creative solutions, such as adapting preparation methods [48] and reconfiguring logistics [9, 55, 63, 67] to support school food implementation. The review suggests that they are not passive recipients of policy but active problem solvers whose expertise should inform future policy.

Another significant barrier identified in this review was the lack of time and shortage of trained school canteen staff, which impacted the implementation of such school food standards. This finding aligns with a systematic review by Morton [75], which found that schools face barriers to implementing health‐related policies due to competing academic priorities and inadequate resources. The review found teachers were stretched thin, with limited time and energy to focus on health initiatives amidst other responsibilities [75]. Similarly, for stakeholders including food service directors, the time‐consuming nature of policy implementation was also reported alongside competing priorities. Availability of training support was also identified as critical to support policy implementation at the school level [70].

To overcome these barriers, the review findings suggest that schools require assistance with implementation and need to be involved in the formulation of nutrition standards of school food policy. The distant perspective and role of food service professionals also need to be considered and included. Their role in the implementation of school meal standards must be viewed as strategic, not just operational. Their desire to be included in the decision‐making processes is evident as many expressed deeper involvement in policy development, decision‐making, and communication [50, 58, 60, 61]. Thus, while food service professionals are key to school food standard implementation, their voices are often excluded from related policy formulation. Collaborative approaches that include all stakeholders can lead to a better understanding of the complex factors influencing implementation [76] and create a sense of ownership and motivation [77]. Adapting policies to different school contexts and allowing for flexibility can also facilitate implementation and the sustainability of nutrition standards for school food. The Diffusion of Innovations theory supports gradual policy introduction to allow stakeholders time to move through the phases of awareness, interest, evaluation, trial, and adoption [78]. It describes how individuals adopt at different rates and identifies key factors that influence adoption, including complexity, compatibility, and perceived advantage. The theory also helps to understand why and how certain innovations are successfully implemented or hindered [78].

One of the most frequently cited facilitators was the combined benefit of both training and partnership/collaboration that came from attending workshops. Training and connections offer tailored support, alleviate concerns, inform practice, and build networks. This finding is consistent with a systematic review by Ponsford et al. [79], which highlighted the importance of ongoing support and resources to enhance “sense‐making” during implementation. The review also found that developing and maintaining organizational relationships and networks can mitigate barriers such as space and procurement challenges by creating co‐ops within districts and sharing resources [80]. A similar approach has been adopted in Australia, where schools share infrastructure and resources through the concept of Community Hubs [81].

## 5 Implications for School Health Policy, Practice, and Equity

The review findings provide valuable insights for researchers, practitioners, and policymakers to develop strategies that address barriers and leverage facilitators to improve the adoption, implementation, sustainment, and scale‐up of nutrition standards for school food. Recommendations for future practice include:

- Policy co‐creation and development: Ensure policies are grounded in practical realities through inclusive and active policy co‐creation, development, and evaluation. By engaging all relevant stakeholders, including food service professionals, it will avoid a top‐down approach. Provide a transition period between policy development and implementation to allow stakeholders to understand, adapt, and pilot the policy before going into effect.
- Financial investment: Provide adequate funding to support the implementation of school meal standards, including ancillary non‐food costs and increased funding for other areas in schools affected by the loss of competitive food sales.
- Adaptation: Carefully consider policy transferability across different school contexts (e.g., school type, geographical locations, and demographics).
- Policy evaluation: Regularly review and update policies to reflect the ever‐changing environment, such as rising food and labor costs, and ensure support is sufficient.
- Monitor implementation: This may incentivize the implementation of school meal standards, promote best practices, and uphold accountability.
- Education: Support schools with necessary resources and adequate training opportunities.
- Future research: Assess the relative importance of determinants and co‐develop and test tailored strategies to improve implementation. More internationally diverse research is required to better understand how implementation barriers and facilitators vary by setting.

## 6 Limitations

While best practices for systematic reviews were followed, the screening of several thousand articles can lead to random errors [82] and inadvertent bias, similar to primary research [83]. The CFIR was not used in the included studies, and the categorization and coding of barriers and facilitators based on the authors' interpretation of the framework may vary depending on the perspectives of others [72]. The one‐dimensional nature of CFIR [84] does not allow for comparisons of policy implementation differences based on factors such as location, school type, food types, phase of implementation, monitoring for compliance, subsidization, and whether standards are mandatory or voluntary. The dominance of US‐based research also limits the generalizability of findings to countries with different school food policies and structures. Most included studies were cross‐sectional, limiting clarity about which factors exert the most significant influence. Despite using a quality appraisal tool, all studies were synthesized equally, potentially undervaluing or overemphasizing evidence from certain studies.

## 7 Conclusion

If the implementation of school food standards is a policy priority throughout the world, then proper resourcing in the form of support, funding, and resources must be provided. Including and centering food service professionals' perspectives is key to successful policy implementation and enables a shift from top‐down implementation to collaborative co‐design. Drawing on their unique insights provides actionable recommendations that reflect the real‐world implementation needs. The review findings can be used by researchers and practitioners to select and develop strategies to address barriers that hinder implementation and utilize facilitators that assist with implementation efforts. Future research should assess the relative importance of the determinants of implementation highlighted in this review.

## Ethics Statement

The authors have nothing to report.

## Conflicts of Interest

The authors declare no conflicts of interest.

## Data Availability

The data that support the findings of this study are available from the corresponding author upon reasonable request.
